# Protein Phosphatases Decrease Their Activity during Capacitation: A New Requirement for This Event

**DOI:** 10.1371/journal.pone.0081286

**Published:** 2013-12-02

**Authors:** Janetti R. Signorelli, Emilce S. Díaz, Karla Fara, Lina Barón, Patricio Morales

**Affiliations:** 1 Department of Biomedicine, Faculty of Health Sciences, University of Antofagasta, Antofagasta, Chile; 2 Antofagasta Institute, University of Antofagasta, Antofagasta, Chile; University of Iowa, United States of America

## Abstract

There are few reports on the role of protein phosphatases during capacitation. Here, we report on the role of PP2B, PP1, and PP2A during human sperm capacitation. Motile sperm were resuspended in non-capacitating medium (NCM, Tyrode's medium, albumin- and bicarbonate-free) or in reconstituted medium (RCM, NCM plus 2.6% albumin/25 mM bicarbonate). The presence of the phosphatases was evaluated by western blotting and the subcellular localization by indirect immunofluorescence. The function of these phosphatases was analyzed by incubating the sperm with specific inhibitors: okadaic acid, I2, endothall, and deltamethrin. Different aliquots were incubated in the following media: 1) NCM; 2) NCM plus inhibitors; 3) RCM; and 4) RCM plus inhibitors. The percent capacitated sperm and phosphatase activities were evaluated using the chlortetracycline assay and a phosphatase assay kit, respectively. The results confirm the presence of PP2B and PP1 in human sperm. We also report the presence of PP2A, specifically, the catalytic subunit and the regulatory subunits PR65 and B. PP2B and PP2A were present in the tail, neck, and postacrosomal region, and PP1 was present in the postacrosomal region, neck, middle, and principal piece of human sperm. Treatment with phosphatase inhibitors rapidly (≤1 min) increased the percent of sperm depicting the pattern B, reaching a maximum of ∼40% that was maintained throughout incubation; after 3 h, the percent of capacitated sperm was similar to that of the control. The enzymatic activity of the phosphatases decreased during capacitation without changes in their expression. The pattern of phosphorylation on threonine residues showed a sharp increase upon treatment with the inhibitors. In conclusion, human sperm express PP1, PP2B, and PP2A, and the activity of these phosphatases decreases during capacitation. This decline in phosphatase activities and the subsequent increase in threonine phosphorylation may be an important requirement for the success of sperm capacitation.

## Introduction

Fertilization is the process by which two haploid gametes, the sperm and the egg, unite to produce a genetically distinct individual. In mammals, fertilization involves a number of sequential steps, including sperm migration through the female genital tract, sperm penetration through the cumulus mass, sperm adhesion and binding to the zona pellucida, acrosomal exocytosis, sperm penetration through the zona pellucida, and fusion of the gamete plasma membranes [1]. However, freshly ejaculated sperm are not capable of fertilizing an oocyte. First, they must undergo a cascade of biochemical and physiological changes that facilitate the binding and penetration of the sperm into the oocyte. This time-dependent acquisition of fertilization competence has been defined as “capacitation” [2], [3].

Capacitation normally occurs in the female genital tract; however, it can also be achieved *in vitro* by incubating the sperm in an appropriate culture medium. The *in vitro* study of capacitation has shown this process to be a combination of sequential and parallel events, which occur both in the sperm head (preparation for the acrosome reaction) and tail (hyperactivation). Recently, capacitation has been divided into the following processes: a) fast and early events that comprise activation of the vigorous and asymmetric movement of the flagellum, which occurs as soon as the sperm leave the epididymis; cholesterol loss from the plasma membrane [4]; increased membrane fluidity; changes in intracellular ion concentration [5]; and hyperpolarization of the plasma membrane [6]; and b) slow and late events that comprise changes in the pattern of movement (hyperactivation), ability to carry out the acrosome reaction stimulated by a physiological agonist, and phosphorylation of proteins at Tyr [4], [5]. Interestingly, both fast and slow events are centrally regulated by the activation of the cAMP/PKA (protein kinase A) pathway.

Post-translational modifications through the phosphorylation of serine/threonine (Ser/Thr) and/or tyrosine (Tyr) residues by protein kinases (PKs) and/or the dephosphorylation of these residues by protein phosphatases (PPs) have a central role in many cellular processes. Mature sperm are transcriptionally inactive, unable to synthesize new proteins. Therefore, the need for these cells to alter their function through protein phosphorylation/dephosphorylation is higher than that of other cell types. Protein phosphorylation, specifically Tyr phosphorylation, is known to regulate sperm motility and capacitation in many mammalian sperm [5]. There have been many studies on the regulation of protein kinases and Tyr phosphorylation during sperm capacitation. In contrast, there are very few studies on Ser/Thr protein phosphorylation and phosphatase regulation during this process.

Generally, PPs are classified into two families: serine/threonine phosphatases (PPPs) and phosphotyrosine phosphatases [7]. The PPPs family includes PP1, PP2A/PP4/PP6, PP2B, PP5, and PP7 gene subfamilies that share high homology in the catalytic domains but differ in their N- and C-terminal domains [7], [8], [9]. Several PPPs family members are expressed in cells from testis and/or sperm [10], and are involved in sperm motility regulation. PP1 has four catalytic subunit isoforms, which are encoded by three different genes: PPP1α/A, PPP1β/B, and PPP1γ/C. PPP1CC1 (PP1γ1) and PPP1CC2 (PP1γ2) are the alternatively spliced variants generated from the single gene PP1γ. This catalytic subunit interacts with more than 200 types of regulatory subunits [10], [11], which are known as PP1 interacting proteins (PIPs). The PIPs control PP1 activity, subcellular location, and substrate specificity. Although PP1α, PP1β, and PP1γ1 are ubiquitous, PP1γ2 is predominantly expressed in the testis and appears to be the only isoform in sperm. The PP1γ2 isoform has been detected in mouse, hamster, bull, primate, and human sperm. There are studies that suggest that PP1γ2 is involved in the regulation of motility activation and/or hyperactivation in mammalian sperm [12]–[14]. Leclerc et al. [15] reported that PP1 was associated with sperm motility and capacitation in human sperm. PP2A is a heterotrimeric phosphatase and consists of one catalytic subunit and two regulatory subunits known as the A subunit and the B subunit. The presence of PP2A has not been reported in human sperm, but its activity has been measured in human and primate sperm extracts [16]. Regarding its function, it may enhance hyperactivation and tyrosine phosphorylation in rodent sperm [14], [17]. The calcium/calmodulin-dependent phosphatase calcineurin (PP2B) consists of a catalytic subunit A (CNA) and a regulatory subunit B (CNB). CNA contains an N-terminal phosphatase domain followed by a CNB-binding helical domain, a Ca2+-calmodulin-binding motif, and an autoinhibitory element. Calcineurin is inactive alone and only gains phosphatase activity upon association with Ca2+-calmodulin [18]. Calcineurin has been detected in dog, goat, porcine, bovine, and human sperm. In human sperm, calcineurin participates in Ca2+-regulated motility parameters and the dephosphorylation of Ca2+-dependent proteins [19]. PP2B is also associated with the regulation of sperm motility and the acrosome reaction in dog, boar, sea urchin [20], [21], and fowl [22]. Castillo et al. [23] demonstrated the presence and the participation of PP2B in the dephosphorylation of synaptotagmin VI during acrosomal exocytosis in human sperm. A recent report from Battistone et al. [24] strongly suggested that human sperm capacitation requires Ser/Thr phosphatase down-regulation. However, until now there are no reports of direct measurements of phosphatase activity during capacitation.

The aim of the present study was to characterize the role of the Ser/Thr PPs PP1, PP2A, and PP2B during human sperm capacitation. We determined which PPs are present in human sperm; characterized their enzymatic activities during capacitation; and, using various inhibitors, evaluated their role in the capacitation process.

## Materials and Methods

### Ethics statement

The research presented in this manuscript was approved by the Ethics Committee on Scientific Research of the University of Antofagasta. The institutional review board approved the use of all human semen samples described in this study. All donors signed a consent form for the use of their sperm cells for research purposes.

### Reagents and Antibodies

The following reagents were purchased from Sigma Chemical Co. (St. Louis, MO): Nα-tosyl-Lys chloromethyl ketone HCl (TLCK); bovine serum albumin (BSA; A7030); 1,4-diazabicyclo[2.2.2]octane (DABCO); HEPES; dimethylsulfoxide (DMSO); 7-chlortetracycline hydrochloride (CTC); poly-L-lysine hydrobromide; Ponceau red; ethylenediaminetetraacetic acid (EDTA); ethylene glycol tetraacetic acid (EGTA); Hoechst 33258 (H258); Igepal CA-630 (NP-40); phenylmethylsulfonyl fluoride (PMSF); sodium dodecyl sulfate (SDS); leupeptin; calmodulin; and bestatin A. The serine-threonine phosphatase inhibitors okadaic acid, deltamethrin, and calmodulin were obtained from Calbiochem Corporation (La Jolla, CA). Endothall was purchased from Affinity Research Products (Biomol Research Laboratories, Farmingdale, NY). The protein phosphatase inhibitor-2 (I-2) was purchased from New England Biolabs (Ipswich, MA). The chemiluminescence detection kit was purchased from Amersham Pharmacia Biotech (Piscataway, NJ). Immobilon P transfer membrane was obtained from Millipore Corporation (Bedford, MA). The DC Protein Assay was obtained from BioRad Laboratories, Inc. (Hercules, CA). Vectashield was purchased from Vector Laboratories, Inc. (Burlingame, CA).

The following primary antibodies were used: anti-phosphothreonine (42H4) n° 9386 (Cell Signaling, Danvers, MA); anti β-tubulin (Developmental Studies Hybridoma Bank, Iowa); anti PP1γ (C-19) (Santa Cruz Biotechnology Inc., Dallas, TX); anti-PP2Bα (482–494); anti PP2A/A (PR65, 7–19) (Calbiochem Corporation, La Jolla, CA, USA); and PP2A antibody sampler kit, n° 9780 (Cell Signaling, Danvers, MA). The following secondary biotinylated antibodies were used: goat anti-rabbit, goat anti-mouse, and rabbit anti-goat (Chemicon International, Temecula, CA).

### Culture media

The basic medium used for all the experiments was a modified Tyrode's medium (117 mM NaCl, 19 mM sodium lactate, 2.5 mM glucose, 8.6 mM KCl, 2.4 mM CaCl2x2H2O, 0.25 mM sodium pyruvate, 0.49 mM MgCl2x6H2O, 0.36 mM NaH2PO4xH2O, 25 mM NaHCO3, 70 µg/ml of penicillin and streptomycin, phenol red, and 2.6% (wt/vol) BSA. This medium was called Capacitating Medium (CM). The Non-Capacitating Medium (NCM) was similar to CM, except for the lack of BSA and NaHCO3, and the addition of 10 mM NaCl and 10 mM HEPES to maintain osmolarity and pH [25]. The Reconstituted Capacitating Medium (RCM) was NCM supplemented with 2.6% BSA and 25 mM NaHCO3. The pH of all media was adjusted to 7.4–7.45 before use.

### Sperm extract preparation

Freshly ejaculated sperm from healthy volunteers were obtained by masturbation after 2–5 days of sexual abstinence. All semen samples were normal according to World Health Organization criteria [26]. Motile sperm were separated using a double Percoll gradient, as described previously [27]. Briefly, aliquots of semen were layered over the upper step of the Percoll gradient and centrifuged at 300×g for 20 min. The pellet was then resuspended in 10 ml of CM or NCM and centrifuged again at 300×g for 10 min. Finally, the sperm cells were resuspended in the appropriate medium at a concentration of 10×10^6^ cells/ml and incubated at 37°C and 5% CO_2_ in air for different periods between 0 and 18 h, depending on the experimental condition. At the end of each incubation time, sperm aliquots were washed twice in PBS (137 mM NaCl, 2.7 mM KCl, 1.5 mM KH_2_PO_4_, 4.3 mM Na_2_HPO_4_, pH 7.4) by centrifuging at 800×g for 5 min. The resulting washed sperm pellet was resuspended in homogenization buffer (50 mM HEPES, 10% glycerol, pH 7.4) at a concentration of 30×10^6^ cells/ml [28], [29] and maintained at −20°C until use.

### CTC Assay

The sperm capacitation status was assessed using the CTC fluorescence assay method, as described previously [30], [31]. Briefly, the CTC solution was prepared on the day of use and contained 750 µM CTC in a buffer of 130 mM NaCl, 5 mM cysteine, and 20 mM Tris-HCl, pH adjusted to 7.8. This solution was kept wrapped in foil at 4°C until use. A 10 µl aliquot of a sperm suspension treated with 10 µg/ml H258 was incubated at 37°C for 10 min; 10 µl of CTC stock solution was then rapidly added, followed within 30 sec by 2 µl of 2% glutaraldehyde in 1 M Tris buffer (pH 7.8). Twenty µl of this suspension was placed on a slide: once it was dried, a drop of DABCO mounting medium was carefully mixed in to retard fading of the fluorescence, and a cover slip was placed on top. Cells were assessed for their living/dead state using H258, as described previously [32]. In each sample, 200 live cells were assessed for CTC staining patterns, and in all cases, the proportion of dead cells was very low. There were three main patterns of CTC fluorescence that could be identified: F, with uniform fluorescence over the entire head, was characteristic of non-capacitated, acrosome intact cells; B, with a fluorescence-free band in the postacrosomal region, was characteristic of capacitated, acrosome-intact cells; and AR, with dull or absent fluorescence over the sperm head, was characteristic of capacitated, acrosome-reacted cells [31], [32]. At all three stages, bright fluorescence on the midpiece could be seen.

### SDS-PAGE and Immunoblotting

SDS-PAGE was performed using 12% gels according to the method of Laemmli [33]. Briefly, 30×106 cells/ml were resuspended in radioimmunoprecipitation (RIPA) lysis buffer (containing 150 mM NaCl, 50 mM Tris, 2 mM EDTA, 1% SDS, 1% sodium deoxycholate, 1% NP-40, 1 mM PMSF, 10 mg/ml leupeptin, 10 mg/ml bestatin A, and 10 mg/ml aprotinin, pH 7.4). The sperm suspension was sonicated with six 60 W bursts for 30 sec each, followed by centrifugation for 30 sec at 14000×g in a Beckman microcentrifuge to remove nuclear and flagellar material. One aliquot of each sperm extract was used for protein determination with the DC protein assay, using BSA as protein standard. The other aliquot was boiled for 5 min with sample buffer (500 µM Tris-HCl, 10% SDS, 30% glycerol, 0.5% β-mercaptoethanol, and 0.5% bromophenol blue, pH 6.8) and then immediately stored on ice. Twenty micrograms of protein was loaded in the gel. Electrophoretic transfer of proteins to polyvinylidene fluoride (PVDF) membranes in all experiments was carried out according to the method of Towbin et al. 1979 [34], at 150 V for 1 h at 4°C. Transfer was monitored by Ponceau red stain. The membrane was then blocked (5% BSA and 3% milk in Tris-buffered saline [TBS]-Tween 20 [0.1% v/v, TBS-T]; 0.5% milk and 0.5% BSA in TBS-T; 3% BSA and 2% milk in TBS-T; or 5% BSA and 5% milk in TBS-T, depending on the primary antibody), washed six times, and incubated with primary antibody at 4°C overnight. Blots were then washed six additional times and incubated with the appropriate biotinylated secondary antibody for 1 h at room temperature. The membrane was washed again, as described above, and then the phosphorylated proteins were detected using an enhanced chemiluminescence kit (Amersham Corp., Sydney, Australia) according to the manufacturer's instructions. Prestained protein standards with a molecular mass range of approximately 250–10 kDa were used. Finally, the image analysis system Image 1.42j was used to quantify the changes in intensity of various bands.

### Indirect immunofluorescence

Aliquots of sperm cells (2×10^6^ cells/ml) were gently smeared onto a microscope slide coated with 0.1 µg/ml of poly-L-lysine and allowed to air dry. Slides were fixed in 4% paraformaldehyde in PBS for 15 min, followed by 3 washes with PBS and permeabilization with 0.1% Triton X-100 for 15 min at room temperature. Then, the slides were incubated for 1 h with a blocking solution containing 2% glycine, 1% BSA, and 50 mM NH_4_Cl in PBS. The slides were then washed 3 times and incubated overnight with different antiphosphatase antibodies at 4°C. Following incubation, the slides were rinsed with PBS and incubated for a further 1 h with goat anti-rabbit antibody conjugated to Alexa Fluor® 633 or 555. For each primary antibody used, a control slide in which the cells were incubated only with the second antibody was processed and analyzed. Slides were washed with PBS and mounted with Vectashield mounting solution. Slides were assessed by epifluorescence ultraviolet microscopy at 630 and 570 nm wavelengths with 100×oil objective lens magnification to determine the localization of detected phosphatases.

### Protein phosphatase activity assay

Sperm aliquots of 20×106 cells/ml were centrifuged at 800×g for 35 sec, and the pellet was rinsed once with homogenization buffer A (Tris 10 mM, pH 7.4, 1 mM EDTA) [13] and manually homogenized with equal parts of buffer A and buffer RIPA and then centrifuged at 800×g for 35 sec at 4°C. Individual supernatants were subjected to phosphatase assays on the day of sample collection.

For PPs activity assays, we used a non-radioactive method, the “Rediplate 96 EnzChek Serine/threonine Phosphatase assay kit” (R33700) from Molecular Probes, according to the manufacturer's instructions. Briefly, 50 µl of sperm lysate was added to each well that contained the Ser/Thr phosphatase substrate (6,8-difluoro-4-methyl-umbelliferyl phosphate, DIFMUP) [35] previously solubilized with 50 µl of a specific phosphatase reaction buffer. The microplate was incubated for 5 min at 37°C, and the phosphatase activity was measured in a microplate fluorometer, Ascent Fluoroskan (Thermo Scientific), using a 355/455 excitation/emission spectrum. To measure PP1 the reaction buffer was optimized by adding 2 mM DTT and 200 µM MnCL2. To measure PP2A the reaction buffer was optimized by adding 1 mM NiCl2. To measure PP2B the reaction buffer was optimized by adding 10 µg/ml calmodulin and 1 mM NiCl2 [36]–[38]. In addition, the activity of each of the three phosphatases was evaluated in the presence of inhibitors of the other two phosphatases. To inhibit PP1 we used 10 nM OA or 40 µg/ml I-2 [8]; to inhibit PP2B we used 0.1 µM deltamethrin; and to inhibit PP2A we used 0.1 nM OA or 90 nM E.

### Statistics

The kinetics of capacitation and phosphatase activity were analyzed by a one-way analysis of variance with Tukey's post test. Capacitation data in CM, NCM or RCM were analyzed by a two-way analysis of variance with Bonferroni correction. Other data were analyzed by one-way analysis of variance and the Student-Newman-Keuls multiple comparison test for unequal replicates using the Prism program. A difference of P<0.05 was considered significant. All data are presented as mean values+SEM.

## Results

### Presence of phosphatases in human sperm

**Figure 1:**
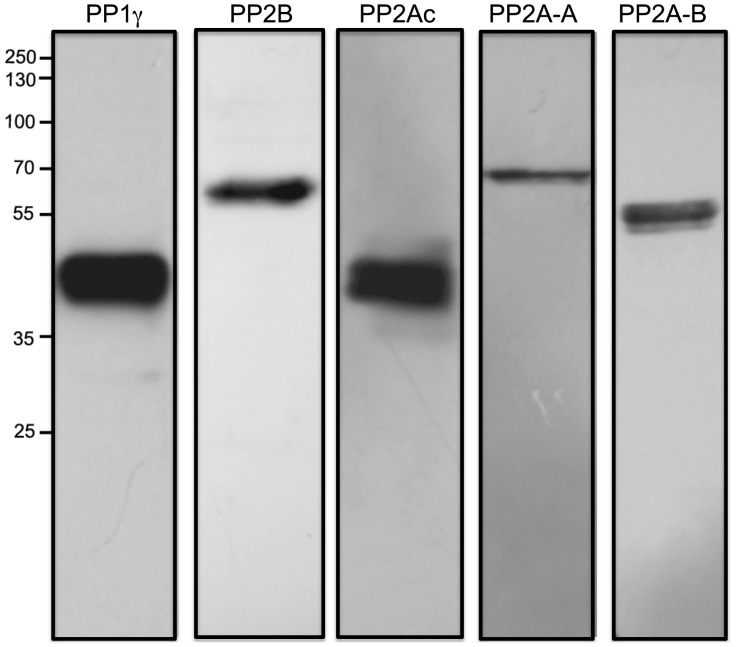
Presence of PP1γ, PP2B, and PP2A in human sperm. Twenty-microgram samples of sperm extracts were subjected to SDS-PAGE and then transferred onto a PVDF membrane. The membrane was then probed with anti PP1γ (PP1γ), anti-PP2Bα (PP2B), and with an antibody sampler kit anti PP2A (PP2Ac, PP2A-A, PP2A-B). The western blot depicted is a representative experiment repeated at least three times. Molecular mass is indicated at the right.

The presence of PP1γ in human sperm was detected using a polyclonal antibody that recognizes the C-terminal end of the PP1γ1 and PP1γ2 isoforms of human origin, which have a molecular mass of ∼39 kDa (Figure 1). To detect the presence of PP2B, we used an antibody that recognizes the catalytic subunit isoform α of the enzyme (CNA, 60 kDa) (Figure 1). These results confirm the presence of PP1γ and PP2B in human sperm [12], [19], [23]. The presence of PP2A was evaluated using a commercial kit that contains 3 monoclonal antibodies that recognize different subunits of the enzyme (Figure 1). One antibody recognizes the α and β isoforms of the PP2A catalytic subunit (PP2Ac, 38 kDa); a second antibody recognizes isoforms α and β of regulatory subunit A or PR65 (PP2A-A, 62 kDa); and the third antibody recognizes regulatory subunit B, specifically the PR55 subunit belonging to the family B (PP2A-B, 52 kDa). The latter antibody does not cross-react with other B subunits belonging to the families B′, B′, or B″′.

**Figure 2:**
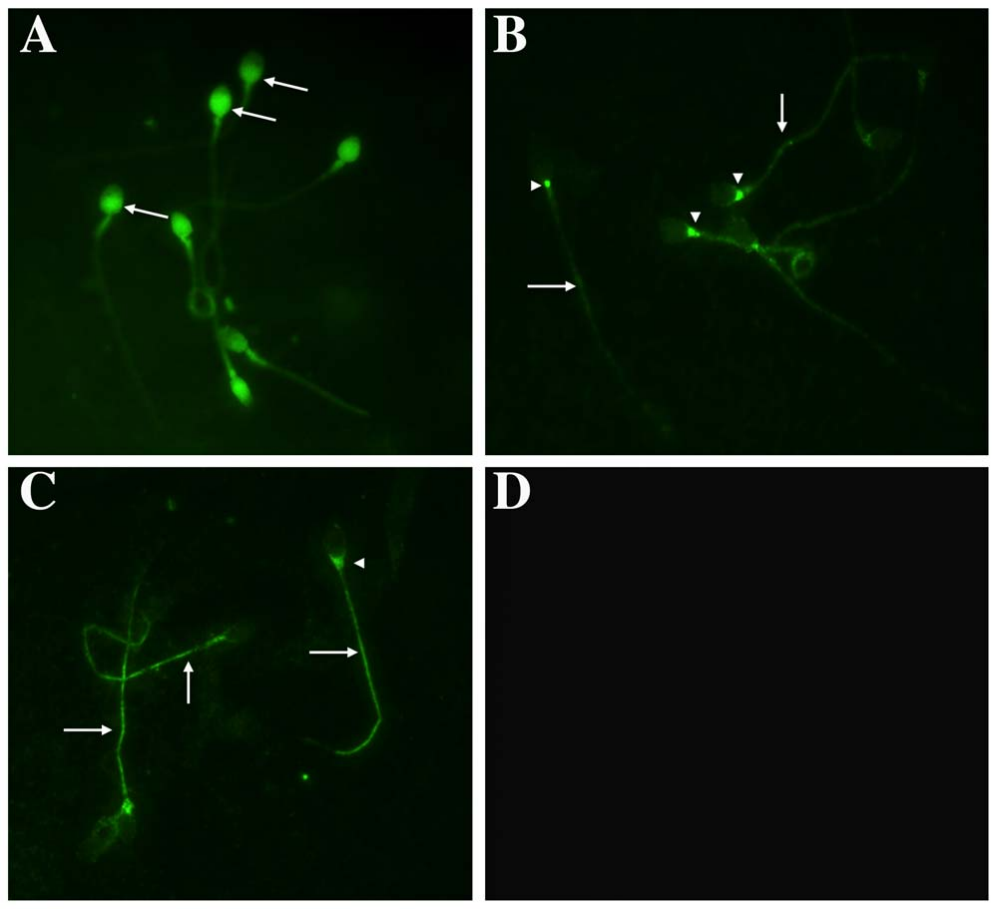
Indirect immunofluorescence of PP1γ, PP2B, and PP2A location in human sperm. Aliquots of sperm selected and re-suspended in NCM were smeared on glass slides, fixed with paraformaldehyde, permeabilized with Triton X-100, and treated overnight with specific anti phosphatase antibodies. Then, samples were treated with Alexa Fluor®633-conjugated anti-rabbit immunoglobulin or Alexa Fluor®555-conjugated anti-goat immunoglobulin. (A) Presence of PP1γ; (B) Presence of PP2B; (C) Presence of PP2A; and (D) negative control. Each micrograph (600×) is representative of three replicates in each of which at least 100 sperm were analyzed.

Given that PP1, PP2B, and PP2A are present in human sperm, we assessed their location by indirect immunofluorescence using the same antibodies as for the western blot assay, except for PP2A, for which we used an antibody obtained from Calbiochem that recognizes the regulatory subunit A, PP2A-A, of 65 kDa (Figure 2). PP1γ was present in the post-acrosomal region and part of the neck region (Figure 2A). In addition, PP1γ was present in the middle and principal piece of the flagellum. Regarding PP2B, in the vast majority of the sperm analyzed, the label was present along the tail (Figure 2B, arrows). In addition, the sperm in this group presented staining at the base of the head, mainly as a dot in this area (Figure 2B, arrowhead). PP2B was also present at the periphery of the head, mainly in the anterior portion (this pattern is not shown in the photo). Regarding PP2A, this enzyme was present in different regions of the sperm (Figure 2C). In the majority of the sperm analyzed, PP2A was present along the tail and at the base of the head. As a positive control, we used an antibody against β-tubulin, which is present in human sperm in the neck and the principal piece of the tail and, in some cases, in the equatorial portion of the head [39] (data not shown). As a negative control, experiments with secondary antibody only were carried out (Figure 2D).

### Effects of phosphatase inhibitors on sperm capacitation

There are very few studies regarding the function of Ser/Thr PPs during capacitation. To understand their role, we incubated the sperm with different inhibitors and evaluated the appearance of sperm with the B pattern. First, we determined the optimal concentration of phosphatase inhibitors to be used. Sperm were resuspended in capacitating medium (CM) and incubated for 1 h in the presence of different concentrations of inhibitors. The inhibitors were used at the IC50 (as described for other cell types) and at concentrations 10 times lower and 10 times higher. To determine the effect of PP1, we used okadaic acid (OA, IC50 10 nM) (Figure S1); to evaluate the effect of PP2B, we used deltamethrin (D, IC50 0.1 nM) (Figure S2); and to determine the effect of PP2A, we used endothall (E, IC50 90 nM) (Figure S3), and OA (IC50 0.1 nM) (Figure S4), respectively [40], [41]. Endothall is a structurally distinct phosphatase inhibitor that is much more potent against PP2A (IC50 90 nM) than PP1 (IC50 5 µM) [40]. Additionally, we used an inactive analog of E, dimethylendothall (DME) at the IC50 of E (Figure S4). It is important to mention that none of the inhibitors at the concentrations used affected the motility or the viability of the sperm; in all cases, the motility and viability were over 80% and similar to the control with DMSO (data not shown). All concentrations used produced a very rapid increase in the percentage of capacitated sperm, measured as the percentage of cells exhibiting the B pattern. The largest effect was observed between 1 and 15 min of incubation and at the concentration that corresponds to the IC50 of each inhibitor (Figures S1, S2, S3, S4). DME did not affect the percentage of capacitated sperm, indicating that the increase in the B pattern after treatment with E was specific (Figure S4).

**Figure 3:**
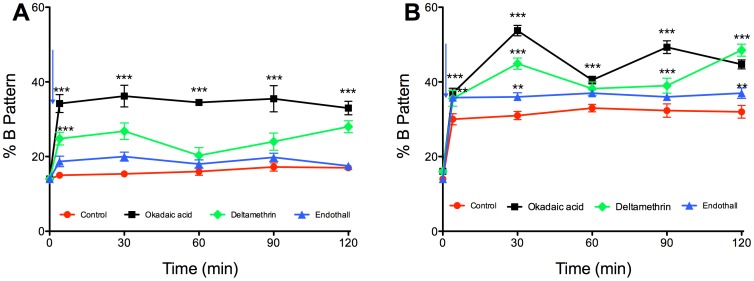
Effect of protein phosphatase inhibitors on human sperm capacitation. Human sperm were incubated in NCM (A) or RCM (B) for 120 min, and the capacitation pattern was evaluated using the CTC assay, as described in the Materials and Methods. The inhibitors used were okadaic acid (black squares), deltamethrin (green diamonds), and endothall (blue triangles). Results were obtained from five different donors and were expressed as the mean±SEM of the percentage of B pattern. The arrow indicates the addition of BSA and bicarbonate. * p<0.05; ** p<0.005; *** p<0.001.

In the following experiments, the effect of each inhibitor was tested on sperm resuspended in either non-capacitating medium (NCM) (Figure 3A) or in reconstituted capacitating medium (RCM) (Figure 3B). Sperm recovered and incubated in NCM exhibited a basal percentage of capacitated cells of 14±0.5%, which did not vary significantly during incubation (Figure 3A). When we added 10 nM OA or D to sperm incubated in NCM, the percentage of cells with the B pattern increased rapidly and significantly at 1 min of incubation, reaching a maximum of 34±2% with OA and 25±1% with D. On the other hand, the addition of E to sperm in NCM did not increase the percentage of cells with the B pattern in relation to the control (Figure 3A). Regarding sperm resuspended and incubated in RCM, the percentage of cells with the B pattern increased significantly and reached a maximum of 33±2% at 120 min of incubation (Figure 3B). When the inhibitors were added to sperm resuspended in RCM, there was a significant increase in the percentage of cells exhibiting the B pattern (Figure 3B). Thus, sperm incubated with 10 nM OA exhibited a percentage of capacitated sperm of 37±2% after 1 min and of 47±2% at the end of incubation. Sperm incubated with D reached a value of 36±2% capacitated cells after 1 min, which continued to grow until reaching a maximum of 49±0.6% at 120 min. In sperm incubated with E, the percentage of capacitated cells increased quickly and significantly and remained constant throughout the incubation period (36±0.9%) (Figure 3B).

### Enzymatic activity of PP1, PP2B, and PP2A during capacitation

**Figure 4:**
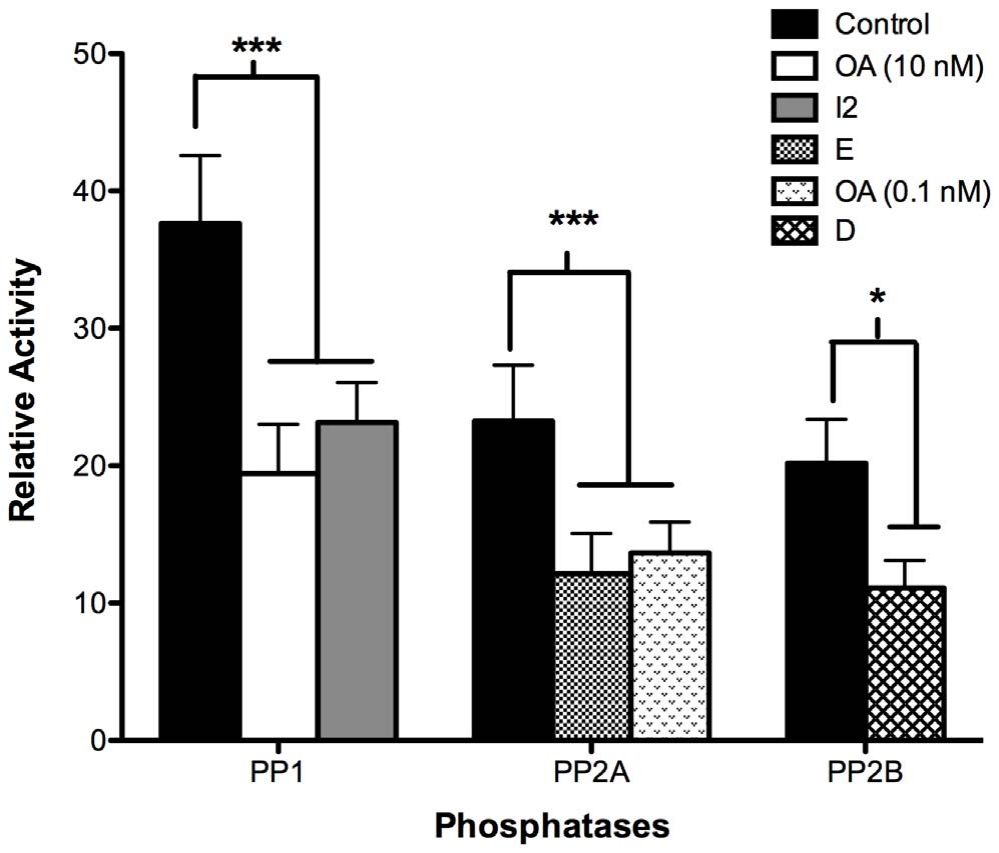
Effect of phosphatase inhibitors on PP1, PP2A, and PP2B enzymatic activity. Sperm were selected and re-suspended in NCM. Each phosphatase activity was measured in sperm extracts as indicated in Materials and Methods in the absence of inhibitors (black bars) and then in the presence of 10 nM okadaic acid (white bar); 40 µg/ml I2 (grey bar); 90 nM endothall (dotted bar); 0.1 nM okadaic acid (light grey bar); or 0.1 nM deltamethrin (striped bar). Results were obtained from five different donors and are expressed as the mean±SEM of forty measurements every 2 min. * p<0.05; ** p<0.005; *** p<0.001. The enzymatic activity is expressed as relative activity.

In the previous experiments, we observed that the inhibitors caused an increase in the percentage of sperm depicting the B pattern. Next, we evaluated the effect of the inhibitors on the enzymatic activity of the PPs. Sperm in NCM were treated with the different inhibitors at their IC_50_ and analyzed immediately (Figure 4). The results indicate that there was a significant decrease in the activity of each phosphatase in the presence of their respective inhibitors. Thus, the activity of PP1 was inhibited by 48±1% and 43±1% in the presence of 10 nM OA and 40 µg/ml I-2, respectively; the activity of PP2A was inhibited by 49±1% and 50±1% in the presence of 90 nM E and 0.1 nM OA, respectively; and the activity of PP2B was inhibited by 53±1% in the presence of 0.1 nM D (Figure 4). These results confirm that the inhibitors used were effective in blocking the activity of the PPs and, therefore, allowed us to ensure that their effect on capacitation was due to an inhibition of the enzyme activities. Treatment with 10 nM OA also inhibited the activity of PP2A (data not shown).

**Figure 5:**
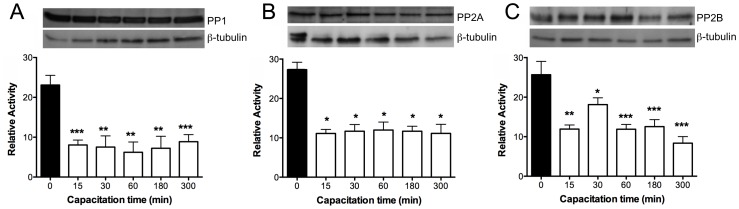
Specific enzymatic activity of PP1 (A), PP2A (B), and PP2B (C) during human sperm capacitation. Sperm were selected and re-suspended in NCM and the PPs activity was measured immediately (black bar, T0). Then, sperm aliquots were resuspended in RCM and incubated at 37°C and 5% CO2 for up to 300 min. Different aliquots were taken at the indicated times and the PPs activity measured as indicated in Materials and Methods (white bars). Results were obtained from four different donors and are expressed as the mean±SEM of forty measurements every 2 min. * p<0.05; ** p<0.005; *** p<0.001. The enzymatic activity is expressed as relative activity. In the insert, the upper panel represents the expression of PP1 (A), PP2Ac (B), and PP2B (C) during the corresponding capacitation times; the lower panel corresponds to the load control with anti β-tubulin antibody.

These results led us to postulate that during capacitation there must be a decrease in the activity of these PPs, which may have a permissive role in this phenomenon. Therefore, we measured the phosphatase activity during capacitation and observed that the activity of each phosphatase was higher in non-capacitated sperm than in capacitated sperm (Figure 5). After 15 min of incubation, the activity of PP1, PP2B, and PP2A decreased drastically and remained low during the 5 h of capacitation (Figure 5). We also evaluated the expression of these enzymes during capacitation by western blotting (Figure 5 insert). The results indicate that there were no changes in the levels of expression of each phosphatase during capacitation, which suggests that the decrease in activity was through another mechanism of regulation, such as phosphorylation and/or breakdown of endogenous activators present in the sperm. It is important to note that the enzymatic activity at 0 min corresponds to the activity of non-capacitated sperm. To verify that the decrease in enzymatic activity that occurred was specifically associated with capacitation, sperm were incubated in NCM for 3 h, and the enzymatic activity was evaluated at the beginning and at the end of the incubation. The results indicate that the enzymatic activity of each phosphatase was similar at both times and suggest that the enzymatic activity does not change depending on the incubation time but depending on the incubation condition (data not shown).

### Protein phosphorylation of Thr residues during sperm capacitation

**Figure 6:**
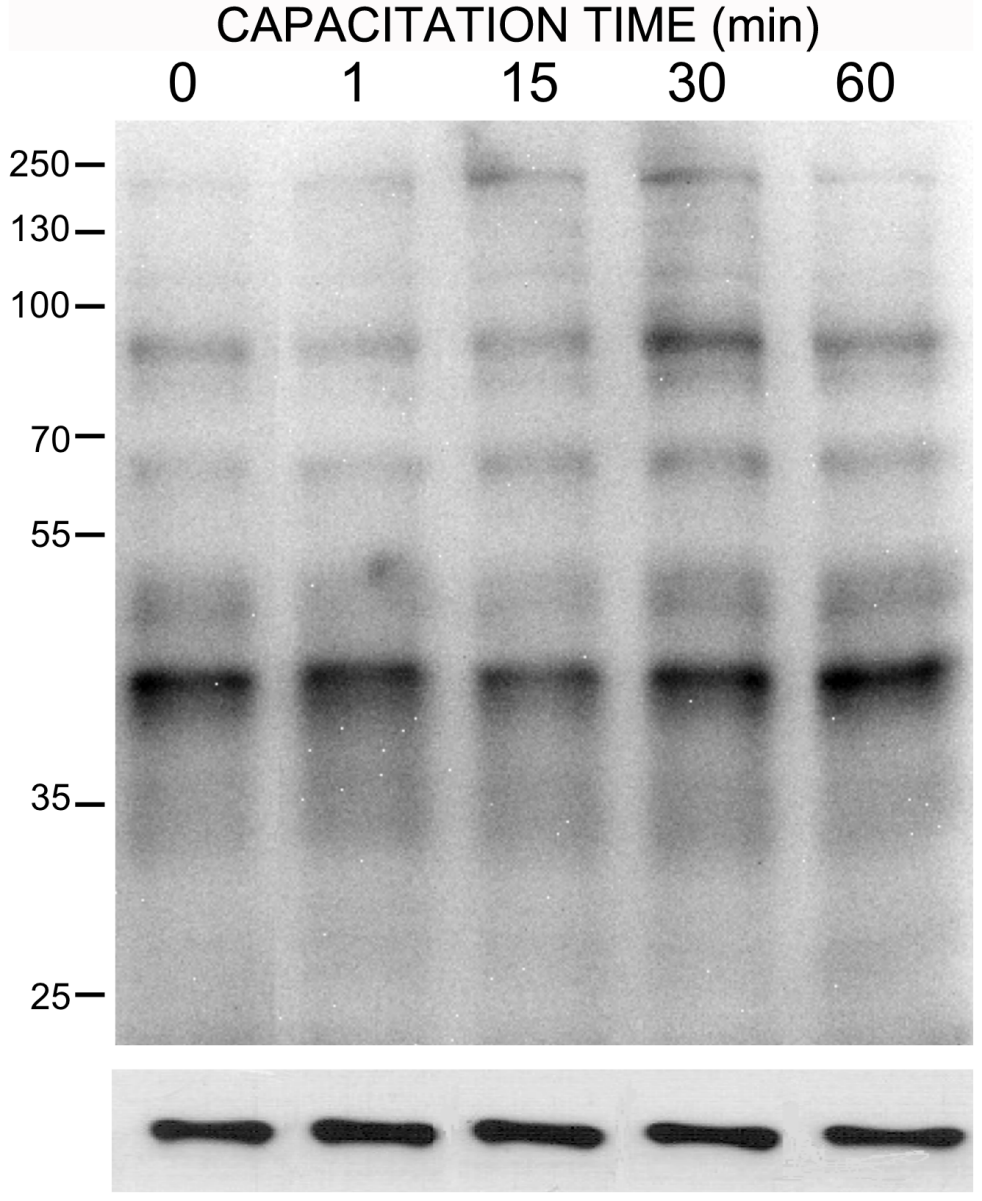
Phosphorylation of threonine residues of human sperm proteins during capacitation. Human sperm obtained in NCM were then resuspended in RCM and incubated for the indicated times. Then, samples were processed for western blot analysis and labeled with a monoclonal anti-p-Thr antibody. The lower panel indicates load control. The immunoblot shown is representative of a total of three different experiments with three different donors.

There is consensus that one important phenomenon during sperm capacitation is the increase in Tyr phosphorylation. However, there are very few studies concerning the role of Ser/Thr phosphorylation during capacitation [42], [43]. In the next experiments, we evaluated the phosphorylation pattern on Thr residues during human sperm capacitation (Figure 6). Sperm were incubated in NCM or RCM for different periods and then analyzed by western blot. Non-capacitated sperm exhibited several bands phosphorylated at Thr (Figure 6). In accord with the decrease in phosphatase activity during capacitation, several bands (i.e., 47, 54, 73, 99, 135 and 153 kDa) increased their phosphorylation during the first hour of incubation.

### Effect of phosphatase inhibitors on the phosphorylation on Thr residues in human sperm

**Figure 7:**
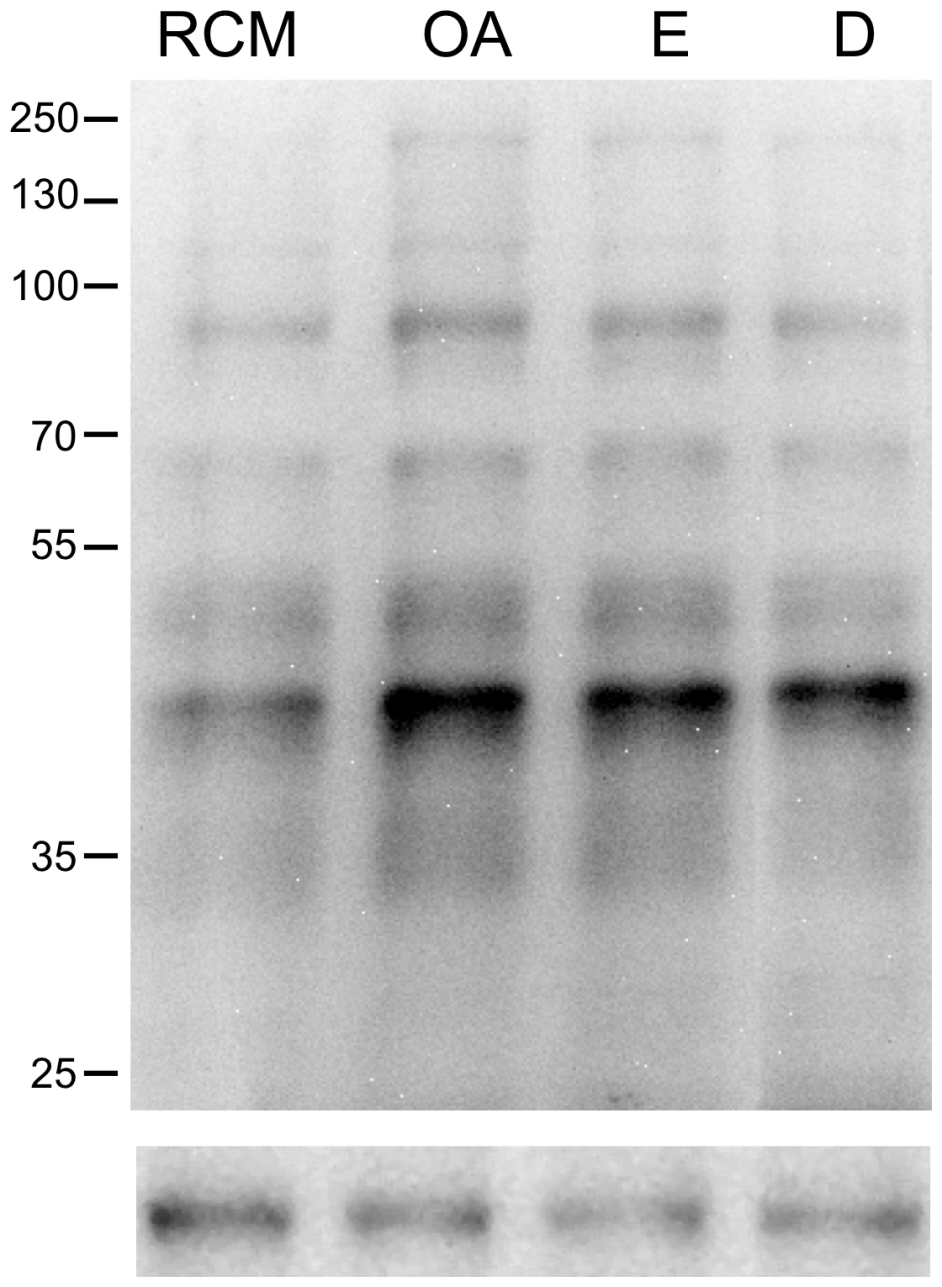
Effect of phosphatase inhibitors on phosphorylation of threonine residues of human sperm proteins. Sperm were selected in NCM and then re-suspended in RCM in the absence (RCM) or in the presence of 10 nM okadaic acid (OA), 90 nM endothall (E), or 0.1 nM deltamethrin (D), for 1 min. Then, the samples were processed for western blot analysis with a monoclonal anti-p-Thr antibody. Molecular mass of selected bands is indicated at the right. The lower panels indicate load control with anti β-tubulin antibody. The immunoblots shown are representative of three different experiments with three different donors.

To determine whether the rapid increase in the percentage of capacitated sperm after phosphatase inhibition correlated with changes in the Thr phosphorylation pattern, we evaluated the phosphorylation pattern in the absence and presence of phosphatase inhibitors. When sperm in RCM were treated with the inhibitors for 1 min, there was an increase in the intensity of several phosphorylated bands (i.e., 47, 53, 73, 99, and 153 kDa) (Figure 7).

## Discussion

The most significant finding of the present work is that Ser/Thr PPs play a physiological role during human sperm capacitation. The evidence in support of this claim was obtained by working with specific inhibitors, measuring the phosphatase enzymatic activities, and performing western blots to detect changes in Thr protein phosphorylation. The experimental evidence that supports this assumption is as follows: 1) three members of the Ser/Thr phosphatase families were detected in human sperm, including PP2A; 2) when any of these three PPs were inhibited, the percentage of sperm depicting B pattern increased rapidly and significantly (≤1 min of inhibitor addition); 3) all of the inhibitors used blocked phosphatase enzymatic activity; 4) the enzymatic activity of these three phosphatases decreased during capacitation; 5) phosphorylation of proteins on Thr residues increased during capacitation; and 6) all of the inhibitors used increased Thr protein phosphorylation in a very fast and dramatic manner.

Currently, there is extensive knowledge regarding the participation of PKs during capacitation, but information about the presence and participation of PPs during this process is scarce. The fact that PKs have an active participation in the capacitation process leads us to hypothesize that PPs may also have an important role. In particular, it has been reported that human sperm incubated in RCM for less than 1 min showed an immediate greater than twofold increase in PKA activity compared with cells suspended in NCM. This increase in PKA activity was followed by an increase in PKA-dependent phosphorylation that begins within 90 sec [44], [45]. Therefore, in the present work, we concentrated our efforts on the study of Ser/Thr PPs. We first confirmed the presence of PP1 and PP2B in human sperm via western blot. PP1 and PP2B have been described in bovine [46], goat [47], canine [20], sea urchin [48], and human sperm [19], [23]. In mammals, the catalytic subunit of PP1 has four isoforms that are encoded by three different genes: PP1α, PP1β, and PP1γ. PP1γ presents two splicing alternative isoforms: PP1γ1 and PP1γ2. Previous studies have described the presence of PP1γ1 and PP1γ2 isoforms in human and monkey sperm extracts [12]. Our results confirm the presence of PP1γ in human sperm. The antibody that we used recognizes the carboxyl-terminal domains of PP1γ1 and PP1γ2 without discriminating between the two isoforms. We also confirmed the presence of PP2B in human sperm by detecting the catalytic subunit of this phosphatase by western blot.

The presence of PP2A has not been described in human sperm. In this work, we present evidence for the presence of PP2A. In particular, we show the presence of the catalytic subunit of PP2A, isoforms α and β (PP2Ac, 38 kDa); the regulatory subunit A or PR65, isoforms α and β (PP2A-A, 65 kDa); and the regulatory subunit B, specifically the PR55 subunit belonging to the B family (PP2A-B, 52 kDa).

Regarding the localization of these PPs, PP1γ appears to be located mainly in the postacrosomal and neck region of human sperm. A less conspicuous label was observed in the middle and principal piece of the flagellum. The subcellular localization of PP1γ has been described in mouse [13], [49], [50], bull [51], and pig sperm [52]. In these species, PP1γ is located in the postacrosomal region, in the equatorial segment of the head, and in the middle and principal piece of the tail. A recent report by Korrodi-Gregório et al. showed a similar pattern [53]. However, our result with OA suggest that this phosphatase may not play a role in sperm motility, as different concentrations of OA did not change the percentage of motile sperm or their motility pattern (data not shown). The localization of PP2B has been described in dog [20] and human [19] sperm. The localization of PP2A has not been reported in sperm of any species. We found that PP2B and PP2A are located in the tail, neck, and postacrosomal region of the sperm. While PP2B and PP2A seem to be localized in the same areas, PP2B is present in the tail as a dotted pattern, and PP2A is present uniformly along the tail. In addition, PP2A is present in the anterior portion of the head in 30% of the sperm, but PP2B was not found in this area. The location more commonly found in our experiments with PP2B match with that described for dog [20] and for human [19] sperm. In addition, that PP2B and PP2A are present in the tail is consistent with their participation in the regulation of sperm motility, which was demonstrated in dog [20] and human [15] sperm, respectively. The latter is the only study that considers the influence of PP2A in motility and/or hyperactivation in human sperm.

To study the function of these PPs during capacitation, we used a PP2B inhibitor, D, and a PP2A inhibitor, E. We also used OA, which is a potent inhibitor of PP1 and PP2A at different concentrations; PP2A is 100 times more sensitive to OA than PP1 [54], [55]. For this reason, when we used 10 nM OA both PPs were inhibited and we could not attribute the observed effect to just one of them. To study PP2A we also used an inactive analog of E, DME. The results with DME allowed us to conclude that the effect of E was specific. First, the effect of the inhibitors was evaluated during capacitation in sperm resuspended in NCM or RCM. In all cases, the maximum effect (approximately 50%) was obtained at the IC50 of each inhibitor. It is important to emphasize that human sperm, unlike those of other species, never reach a percentage of capacitation more than 50–60% [30], [56], [57]. In mouse and hamster, for example, most of the sperm (>90%) became capacitated when they are incubated in an appropriate medium for a few hours [43], [56], [58], [59]. In our study, the maximum percentage of sperm exhibiting the B pattern was in accord with that described by Furuya et al. (1993) and was approximately 50% [60].

In this regard, we observed two related findings that are noteworthy. First, there was a very rapid increase in sperm exhibiting the B pattern when BSA/HCO3- was added to sperm in NCM (to become RCM); second, there was a very rapid increase in capacitation when the PP1 and PP2B inhibitors were added to sperm incubated either in NCM or RCM. In fact, when BSA/HCO3- or the inhibitors were added to the sperm, the percentage of cells with the B pattern increased significantly and rapidly within 1 min of addition. Shortly thereafter, the percent of capacitated cells reached a plateau. After 2 h of incubation, the percentage of capacitated sperm was similar to that of the sperm incubated in CM without inhibitor.

These results are consistent with those reported by Furuya et al. [60] in human sperm. They observed that calyculin A (CL-A) has a fast stimulatory effect on the percentage of capacitated sperm evaluated with the CTC assay. This stimulatory effect increased to a maximum at 15 min (their earliest observation time) and then reached a plateau. After 3 h of incubation there were no significant differences in the percentage of capacitated sperm between the CL-A treated and control groups. A later study by Leclerc et al. [15] showed that human sperm treated with 100 nM OA or CL-A exhibits an increase in the percentage of capacitated cells, evaluated by determining the percentage of acrosome reacted sperm. In their study, the overall effect was the same as that described by us. The same capacitation-accelerating effect was reported for hamster sperm incubated with 100 nM OA [17], [43]. These results confirm that inhibition of the PPs causes an acceleration of the capacitation process and suggests that the PPs should decrease their activity for the capacitation to progress.

In all of the above-mentioned studies, the concentration of inhibitors used was very high (100 nM CL-A [15], [43], [60], 5 µM OA [17]). This fact makes it difficult to discern which of the phosphatases, PP1 and/or PP2A, participates in capacitation. In this report, we used the IC_50_ of each inhibitor, and the results strongly suggest the involvement of both PPs during this process.

Treatment with phosphatase inhibitors provoked a significant and rapid increase in the percentage of sperm depicting the B pattern. To elucidate whether this result was related to an inactivation of the enzymes during capacitation, we then measured the activity of PP1, PP2B, and PP2A during this period. The present results indicate that the activity of each phosphatase significantly decreased during sperm capacitation. This finding strongly suggests that a decrease in the activity of PP1, PP2B, and PP2A may be a requirement for sperm capacitation because non-capacitated cells do not present this reduction in phosphatase activity even if they are incubated for 3 h.

To test whether the loss of phosphatase activity was due to protein degradation, we determined the expression of PP1, PP2B, and PP2A during capacitation by western blotting. There was no variation in the expression of any of the PPs studied for a period of up to 5 h of capacitation. The activity of the PPs is also regulated by events of phosphorylation and dephosphorylation. It has been reported that PP1γ is inhibited through phosphorylation on Thr (Thr320) and that PP2A is inhibited through phosphorylation on Tyr (Tyr307). This effect has been observed in sperm of mouse [14], pig [52], and hamster [61]. These results are consistent with our observations that the enzymatic activities of PP1, PP2B, and PP2A are low during capacitation. Likewise, a recent report from Battistone et al. [24], presented evidence that the decrease in Ser/Thr phosphorylation caused by SKI606 (a Src family kinase inhibitor) was reverted by 100 nM OA. This result indicated to the authors that a Src family kinase might be inhibiting a Ser/Thr phosphatase. Thus, they suggested that PP1γ2 and, to a lesser extent, PP2A are involved in human sperm capacitation[24].

Finally, we were interested in determining whether the inhibition of these PPs produces changes in the phosphorylation pattern on Thr residues. We first evaluated the pattern of phosphorylation on Thr residues during capacitation in sperm incubated for 1 h in RCM. Naz et al. [42] performed similar experiments using human sperm capacitated for 5 h. We observed an increase in protein phosphorylation during the first hour of incubation. The phosphorylated proteins have a molecular mass similar to those described by Naz [42], such as proteins of 47 and 54 kDa (43 and 55 in Naz, 1999) and proteins of 99 kDa (94 kDa in Naz, 1999). Our results show that within the first hour of incubation, there are increases not only in the phosphorylation of proteins of 47 kDa, similar to those found in hamster sperm [43], but also in the phosphorylation of proteins of 54, 73, 99, 120, 136, and 153 kDa.

We also assessed the protein phosphorylation pattern of sperm incubated in RCM in the presence or absence of phosphatase inhibitors. Sperm incubated for short periods of time (≤1 min) in the presence of the inhibitors OA, E, or D exhibit an increase in the phosphorylation of Thr residues of proteins from 42 to 100 kDa. This observation is consistent with the rapid increase in the percentage of capacitated sperm and the rapid decrease in phosphatase activity provoked by the inhibitors. Jha and Shivaji [43], working with hamster sperm, assessed the effects of 200 nM OA on the phosphorylation of Thr residues. They found that proteins of 49 and 63 kDa decreased their phosphorylation at 3 h of incubation and increased their phosphorylation at 5 h of capacitation. Our results show that in the presence of phosphatase inhibitors, including 10 nM OA, there is an increase in the phosphorylation of proteins between 47–100 kDa and 153 kDa. This difference suggests a differential regulation of phosphorylation in sperm of these two species.

**Figure 8:**
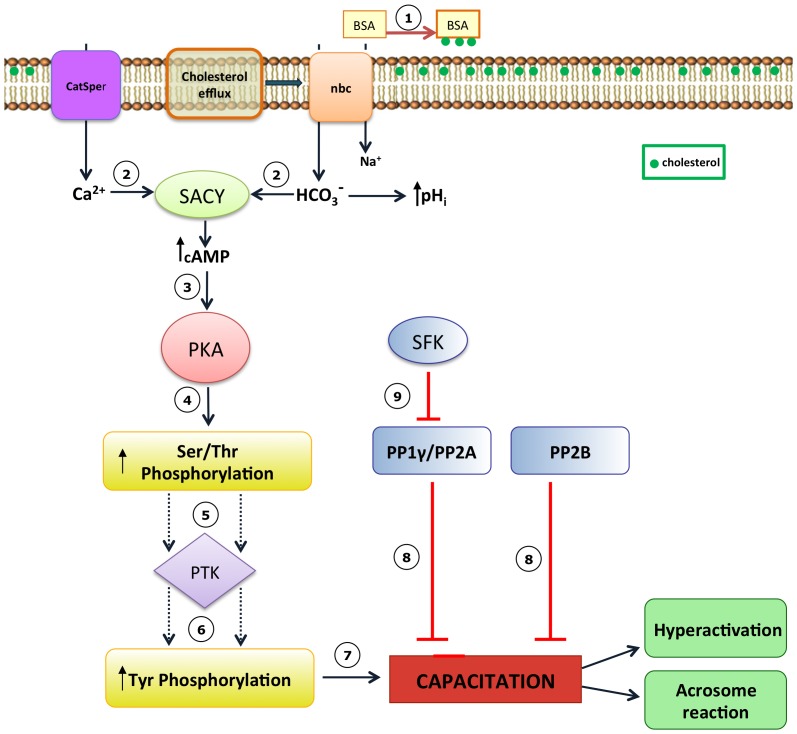
Model for the participation of PP1, PP2A, and PP2B during human sperm capacitation. (1) The first event of capacitation is the efflux of cholesterol mediated by albumin (BSA), (2) causing an increase in membrane fluidity that allows the entry of Ca2+ and HCO3-. These ions activate the soluble adenylyl cyclase (SACY) which increases the level of cAMP (3) and allow PKA activation. (4) PKA increases the phosphorylation of proteins in Ser/Thr residues. (5) This activates tyrosine kinases (PTK), (6) which produce an increase in protein Tyr phosphorylation. (7) Finally, the sperm cells capacitate. (8) Our results indicate that the Ser/Thr phosphatases, PP1, PP2A and PP2B, inactivate during capacitation and this allow sperm to capacitate. (9) Battistone et al. [24] propose that down-regulation of PP1 and PP2A is mediate by SFK and is a parallel pathways to the PKA/cAMP pathway.

In conclusion, we report evidence that suggests that during capacitation the activity of the Ser/Thr PPs PP1, PP2B and PP2A decreases and that this decrease may be required for successful capacitation in human sperm (Figure 8).

## Supporting Information

Figure S1 **Effect of high concentrations of okadaic acid on the capacitation of human sperm.** Sperm resuspended in CM were treated with different concentrations of okadaic acid. The percent of capacitated cells was evaluated at various times. Results were obtained from five different donors and are expressed as the mean±SEM of the percentage of the B pattern. * p<0.05; *** p<0.001.
(TIF)

Figure S2 **Effect of deltamethrin on the capacitation of human sperm.** Sperm resuspended in CM were treated with different concentrations of deltamethrin. The percent of capacitated cells was evaluated at various times. Results were obtained from four different donors and are expressed as the mean±SEM of the percentage of the B pattern. *** p<0.001.
(TIF)

Figure S3 **Effect of low concentrations of okadaic acid on the capacitation of human sperm.** Sperm resuspended in CM were treated with different concentrations of okadaic acid. The percent of capacitated cells was evaluated at various times. Results were obtained from five different donors and are expressed as the mean±SEM of the percentage of the B pattern. * p<0.05; *** p<0.001.
(TIF)

Figure S4 **Effects of endothall and dimethylendothall (DME) on the capacitation of human sperm.** Sperm resuspended in CM were treated with different concentrations of endothall and with 90 nM DME. The percent of capacitated cells was evaluated at various times. Results were obtained from five different donors and are expressed as the mean±SEM of the percentage of the B pattern. *** p<0.001.
(TIF)
