# Supplementary figures and images for: Protein Phosphatases Decrease Their Activity during Capacitation: A New Requirement for This Event
Source: PLoS One. 2013 Dec 2;8(12):e81286. doi: 10.1371/journal.pone.0081286 (PMC3846847; doi:10.1371/journal.pone.0081286)

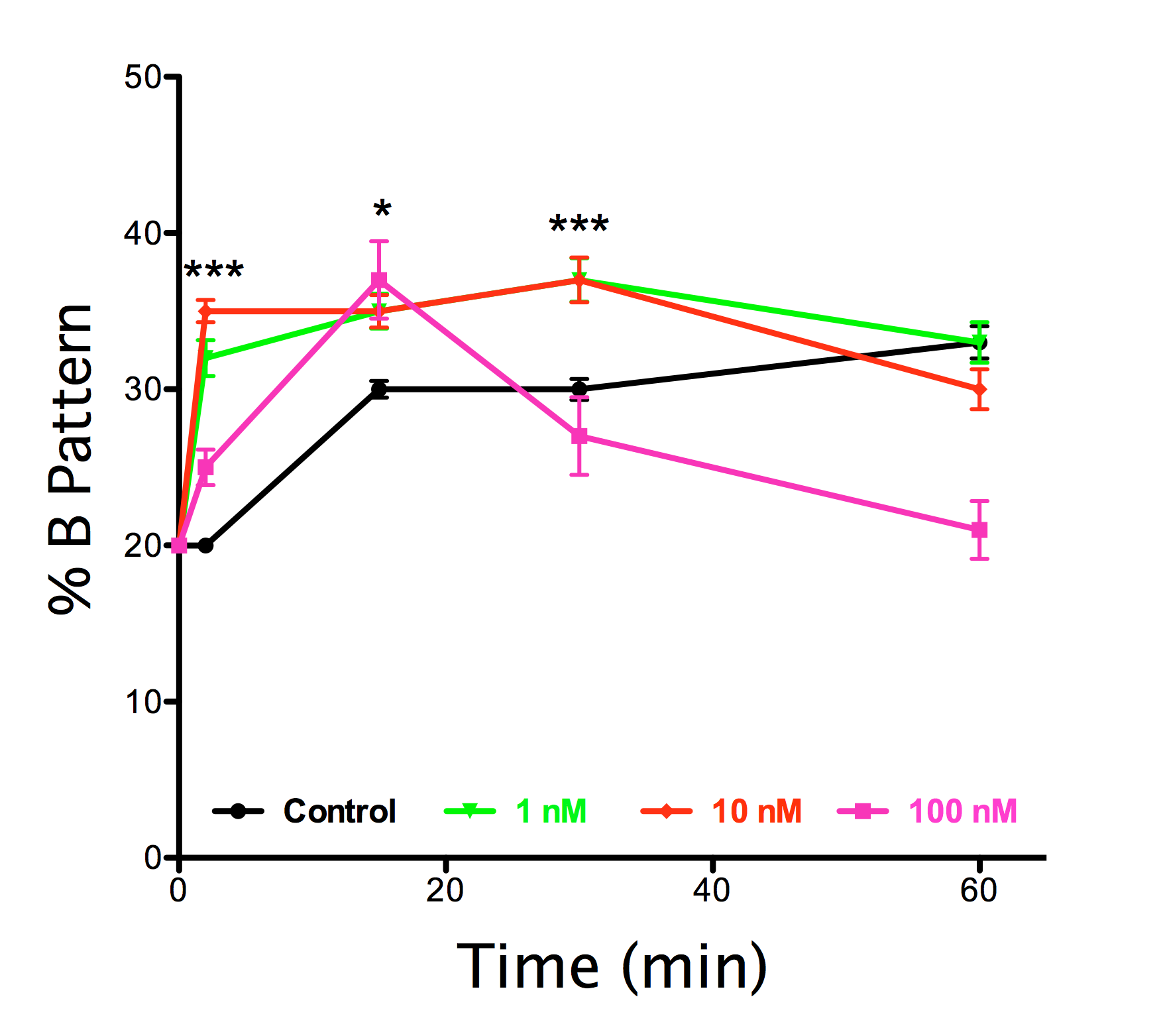

Supplement: Figure S1 — Effect of high concentrations of okadaic acid on the capacitation of human sperm. Sperm resuspended in CM were treated with different concentrations of okadaic acid. The percent of capacitated cells was evaluated at various times. Results were obtained from five different donors and are expressed as the mean±SEM of the percentage of the B pattern. * p<0.05; *** p<0.001. (TIF) [file pone.0081286.s001.tif]

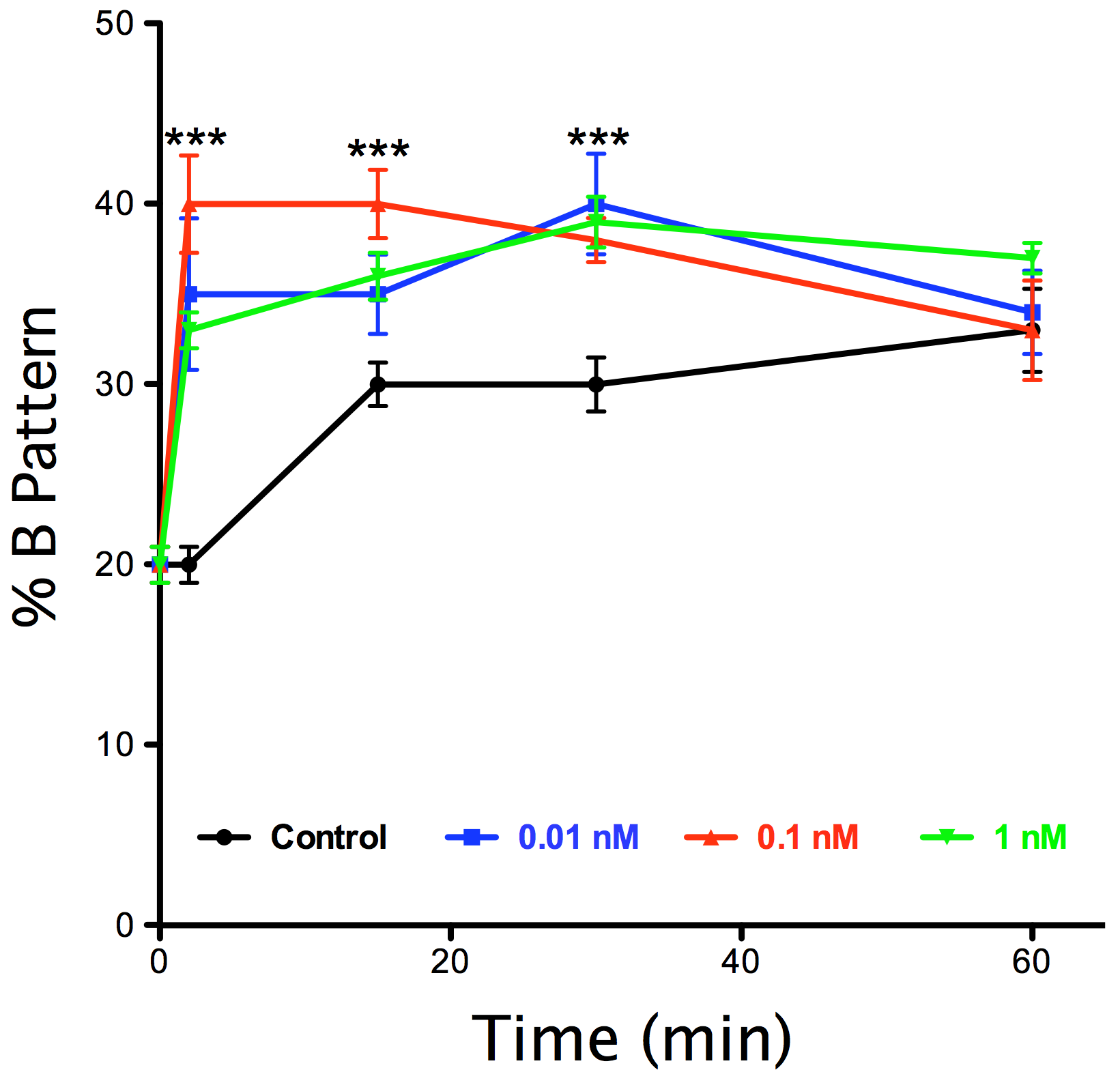

Supplement: Figure S2 — Effect of deltamethrin on the capacitation of human sperm. Sperm resuspended in CM were treated with different concentrations of deltamethrin. The percent of capacitated cells was evaluated at various times. Results were obtained from four different donors and are expressed as the mean±SEM of the percentage of the B pattern. *** p<0.001. (TIF) [file pone.0081286.s002.tif]

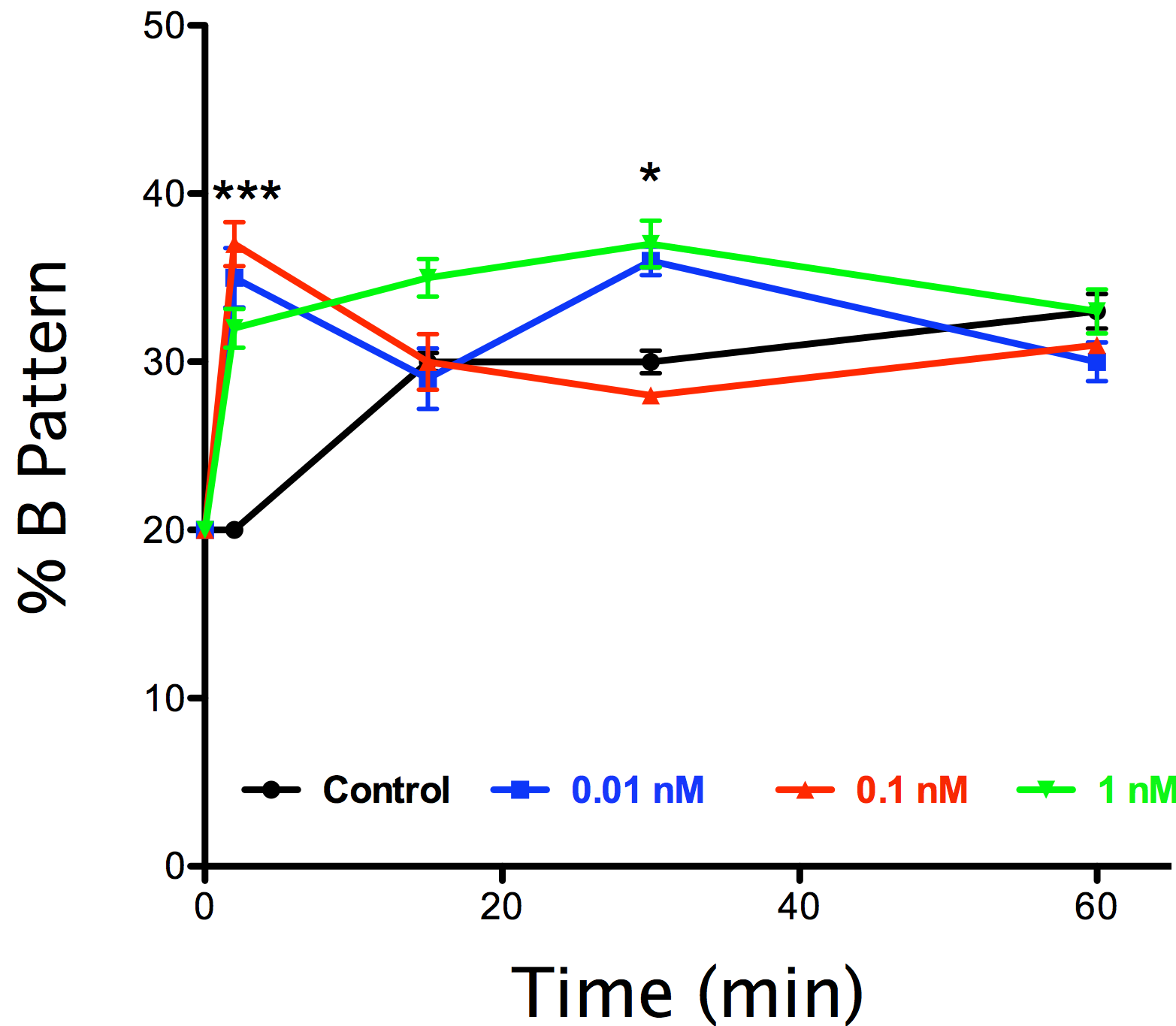

Supplement: Figure S3 — Effect of low concentrations of okadaic acid on the capacitation of human sperm. Sperm resuspended in CM were treated with different concentrations of okadaic acid. The percent of capacitated cells was evaluated at various times. Results were obtained from five different donors and are expressed as the mean±SEM of the percentage of the B pattern. * p<0.05; *** p<0.001. (TIF) [file pone.0081286.s003.tif]

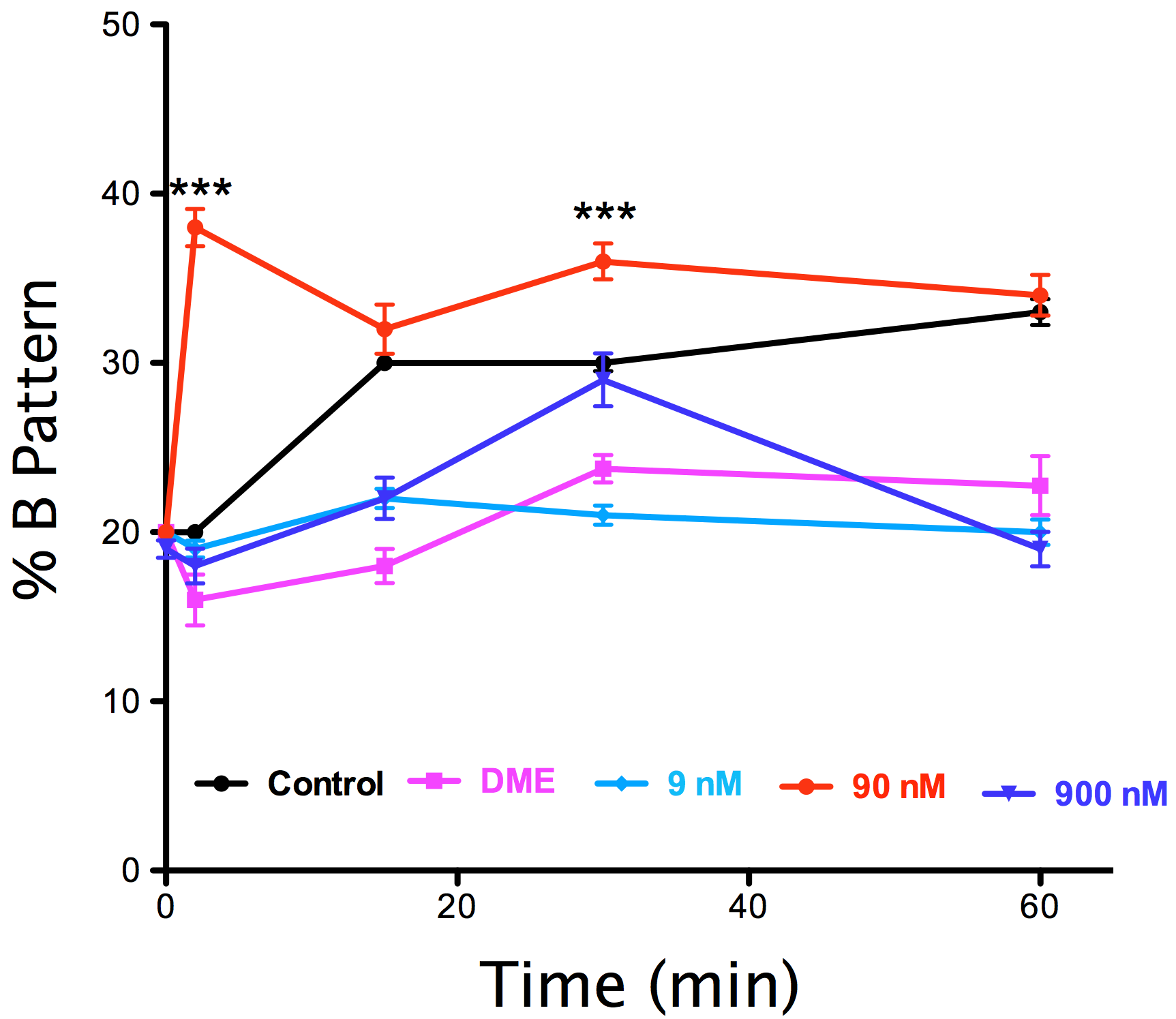

Supplement: Figure S4 — Effects of endothall and dimethylendothall (DME) on the capacitation of human sperm. Sperm resuspended in CM were treated with different concentrations of endothall and with 90 nM DME. The percent of capacitated cells was evaluated at various times. Results were obtained from five different donors and are expressed as the mean±SEM of the percentage of the B pattern. *** p<0.001. (TIF) [file pone.0081286.s004.tif]
